# Canine Length in Wild Male Baboons: Maturation, Aging and Social Dominance Rank

**DOI:** 10.1371/journal.pone.0126415

**Published:** 2015-05-07

**Authors:** Jordi Galbany, Jenny Tung, Jeanne Altmann, Susan C. Alberts

**Affiliations:** 1 Center for the Advanced Study of Hominid Paleobiology, Department of Anthropology, The George Washington University, Washington DC, United States of America; 2 Department of Evolutionary Anthropology, Duke University, Durham, NC, United States of America; 3 Duke Population Research Institute, Duke University, Durham, NC, United States of America; 4 Institute of Primate Research, National Museums of Kenya, Nairobi, Kenya; 5 Department of Biology, Duke University, Durham, NC, United States of America; 6 Department of Ecology and Evolutionary Biology, Princeton University, Princeton, NJ, United States of America; Université de Sherbrooke, CANADA

## Abstract

Canines represent an essential component of the dentition for any heterodont mammal. In primates, like many other mammals, canines are frequently used as weapons. Hence, tooth size and wear may have significant implications for fighting ability, and consequently for social dominance rank, reproductive success, and fitness. We evaluated sources of variance in canine growth and length in a well-studied wild primate population because of the potential importance of canines for male reproductive success in many primates. Specifically, we measured maxillary canine length in 80 wild male baboons (aged 5.04–20.45 years) from the Amboseli ecosystem in southern Kenya, and examined its relationship with maturation, age, and social dominance rank. In our analysis of maturation, we compared food-enhanced baboons (those that fed part time at a refuse pit associated with a tourist lodge) with wild-feeding males, and found that food-enhanced males achieved long canines earlier than wild-feeding males. Among adult males, canine length decreased with age because of tooth wear. We found some evidence that, after controlling for age, longer canines were associated with higher adult dominance rank (accounting for 9% of the variance in rank), but only among relatively high-ranking males. This result supports the idea that social rank, and thus reproductive success and fitness, may depend in part on fighting ability mediated by canine size.

## Introduction

Many primate species show high sexual dimorphism in the canine teeth, with males possessing larger canines than females [1–2]. Although canines sometimes play a role in food processing [3–4], in many species the canine teeth serve a primary function used as weapons in intra-sexual conflicts, and hence evolve under sexual selection [5–11]. Indeed, sexual selection will often favor the development of traits, such as large canines, that aid males in winning contests and thus allow them enhanced access to females [9].

Here, we evaluated sources of variance in canine growth and length in a well-studied wild primate population, because of the potential importance of canines for male reproductive success in many primates. This study contributes to an extremely limited literature on the functional significance of variance in canine size within living primate populations. Indeed, the only previous such study, to our knowledge, is by Leigh and colleagues on a breeding colony of mandrills (*Mandrillus sphinx*) maintained in a semi-natural enclosure [12]. They found a strong relationship between male canine size and reproductive success in mandrills: males that successfully sired offspring had longer canines than males that did not, and only males with canines greater than a threshold length were fathers. Further, the canines became shorter with age through wear, and the canines’ diminishing length during aging corresponded in timing with the male’s diminishing reproductive activity. In other words, canine length (determined by developmental processes in young adulthood and wear during aging) directly correlated with male fitness in mandrills [12].

Yellow baboons (*Papio cynocephalus*), the subjects of this study, live in multimale-multifemale social groups that are characterized by linear dominance hierarchies. Male baboons disperse from the natal social group within a few years of reaching sexual maturity; after dispersal, they typically join a new group and gain a dominance rank position in that group. In baboons and a number of other primates, male dominance rank depends on fighting ability. Male-male contests over dominance rank or access to females may involve both canine displays and injuries from bites, which in turn may result in wounds that occasionally have lethal consequences [13–14]. However, high dominance rank may confer substantial fitness benefits in baboons, as in other primates, which potentially outweigh the risks of fighting (reviewed in [15]). Specifically, high-ranking male baboons are more likely than lower-ranking males to engage in contsortships (mate-guarding episodes), which may last from several hours to a few days. Male dominance rank declines with age in this species after reaching a peak in young adulthood; maximum observed lifespan in Amboseli males is 25 years, but few males live beyond 20 years. However, male baboons that obtain more consortships produce more offspring [16–17], indicating that both fighting and mate-guarding have substantial benefits for male baboons in spite of the costs. Thus, as in the case of mandrills, male baboons are a good system for understanding the relationships between canine length, age, and social dominance rank [16,18].

In the present study we tested three hypotheses related to canine length in wild male baboons. First, we examined patterns of canine growth during the adolescent period, when males are in the process of developing the canines that will later serve as weapons. Previous research has indicated that the timing of tooth eruption may depend on nutritional conditions. For instance, the eruption of upper canines in male baboons occurred at a median age of 76 months in wild yellow baboons in Mikumi National Park, Tanzania, whereas captive male yellow baboons at the Southwest Foundation for Biomedical Research in Texas experienced upper canine eruption at a mean age of 57 months [19–20]. In addition, in Amboseli, Kenya, food-enhanced animals (those that forage part time on refuse from a tourist lodge) gained body mass more rapidly and reached puberty earlier than wild-feeding animals [21], a common finding in primates when food resources are supplemented (reviewed in [22–24]). We lacked data on the timing of tooth eruption *per se*, but we reasoned that we could potentially infer differences in the timing of eruption by examining the intercepts (as well as the slopes) of the regressions of canine length on age in the two feeding conditions. Specifically, we hypothesized that male baboons subsisting on entirely wild foods found in the natural foraging environment would experience later canine eruption, and hence shorter canine length for a given age, than food-enhanced males (Hypothesis 1).

Second, we examined canine wear during aging. Once erupted and grown to their maximum length, upper canines in baboons are continuously worn against the lower canines and the first premolar (P3) teeth. This functional attrition or honing creates wear facets, especially in males, and maintains a sharpened canine tip and edge [25–26]. Unlike the premolars, which are more protected against wear because of their thick enamel, the upper canines have thin enamel and wear down easily [25–26]. Canine honing and wear occur not only during the course of mastication, but also during tooth grinding related to aggression, tension release, and canine displays or “yawns” [25–26]. As in predatory mammals, canine loss or damage results in a complete or partial loss of canine functionality, because severely blunted canine teeth are inferior weapons, even for display purposes [27]. We hypothesized that canine length would be shorter in older adult male baboons than in younger and prime-aged adults, as a result of tooth wear (Hypothesis 2).

Third, we examined the relationship between male canine length and dominance rank. Rank attainment in male baboons coincides with the end of subadulthood and the onset of a period of rapid rise in dominance rank among adult males; once the male has attained an adult rank, he has the opportunity to engage in mate guarding (consortships) [16,18]. As is typical in many baboon populations, male baboons in Amboseli that attain a high dominance rank have higher mating success and sire more offspring than males of lower dominance ranks [16,18]. Thus, if longer canines help males compete for high rank, then both canine growth and wear rates may be important in determining male fitness. We hypothesized that canine length, corrected for age, would predict social dominance rank, an important predictor of reproductive success in male baboons (Hypothesis 3).

## Material and Methods

### Ethics statement

All research reported in the manuscript adhered to the American Society of Primatologists Principles for the Ethical Treatment of Non Human Primates. Research protocols were reviewed and approved in Kenya (Kenya Research Permits MOEST 13/001/C351 Vol. II and MOS&T 13/001/33c/79) and the United States (IACUC A0840903 at Duke University and 1689 at Princeton University).

### Study population and life history data

The Amboseli basin (2°40' south and 1100 meters above sea level) is a semiarid savannah in southern Kenya, near the base of Mount Kilimanjaro in East Africa [28]. The area is populated by yellow baboons (*Papio cynocephalus*) that exhibit some admixture with anubis baboons (*Papio anubis*), which occasionally immigrate into the basin [29–30]. This population has been intensively studied for more than four decades [31]. Detailed longitudinal data on demography, ecology, behavior and physiology are available for a large number of individuals [21,32–33]. The long-term Amboseli baboon data set, in combination with cross-sectional data on tooth condition collected during periodic immobilization projects, made it possible to ask questions about sources of variance in canine growth and length in this population (e.g., [34–35]).

Subjects (*n* = 80) were male members of five social groups of wild-feeding baboons that were darted between 1989 and 2010, and members of one social group of food-enhanced baboons that was darted between 1989 and 1994 (*n* = 5). All individuals in the study groups were recognized on sight and habituated to the presence of human observers. Demographic and life history records were drawn from Babase, the long-term database of the Amboseli Baboon Project. Male dominance rank, a good proxy for fighting ability for males, was determined on a monthly basis for all subjects by assigning wins and losses in dyadic agonistic encounters. Males were considered to win agonistic encounters in which they gave only aggressive or neutral (nonsubmissive) gestures while their opponent gave only submissive gestures [36]. This procedure of assigning wins and losses allowed us to construct square monthly matrices of interactions in which entries below the diagonal (which would represent wins by the lower-ranking animal) were few or zero [18]. Ordinal ranks were then assigned based on an individual’s position in the matrix, so that the highest-ranking male in the group was assigned an ordinal rank of 1, the second-ranking male was assigned an ordinal rank of 2, and so on.

Of the 80 males in this study, 44 had been studied from birth, and their ages were known exactly. For the remaining 36 subjects, ages were estimated based on patterns of growth, maturation and change in physical features over time (see [16]). Four of these 36 subjects were first observed as relatively young juveniles and were then followed for at least 4 years during their transition to adulthood; age estimates for these individuals were based on patterns of growth and maturation during the juvenile and adolescent period, which are well-described in our population [21]. For the 32 individuals whose ages were estimated as adults, age estimates were based on close observation over multiple years (30 of them were resident in our study population for between 2.5 and 19.9 years; two were resident for less than two years). For 24 of these 36 individuals, highly trained field observers assigned error estimates of ± 1 year (i.e., if the age assignment was 9 years, the actual age was estimated to be between 8 and 10 years). For 11 individuals, error estimates were ± 2 years, and for 1 individual the error estimate was ±3 years (Table 1). We have assigned age estimates to immigrant males in this manner for more than 2 decades; most males are assessed multiple times by all the senior field observers.

**Table 1:** Study subjects (N = 5 food-enhanced and 75 wild-feeding male baboons), including feeding condition, age, maturation status, social rank, and canine length.

Animal ID	Feeding	Age (years)	Maturation status	Canine Length (cm)	Social Rank	Birthdate status*	Researcher**
1	Enhanced	5.04	Not yet adult	0.8	10	0	JA
2	Enhanced	5.40	Not yet adult	0.5	17	0	JA
3	Enhanced	5.80	Not yet adult	2.7	8	1	JA
4	Enhanced	6.26	Adult	2.3	10	0	JA
5	Enhanced	6.72	Adult	3.4	4	1	JA
6	wild	5.98	Not yet adult	0.8	12	0	JA
7	wild	6.11	Not yet adult	0.5	11	0	JA
8	wild	6.22	Not yet adult	0.4	15	0	JG
9	wild	6.48	Not yet adult	1.6	17	0	JG
10	wild	6.67	Not yet adult	1.7	14	0	JG
11	wild	6.75	Not yet adult	1.1	9	0	JG
12	wild	6.93	Not yet adult	1.0	10	0	JA
13	wild	7.25	Not yet adult	2.5	19	0	JG
14	wild	7.27	Not yet adult	2.2	8	0	JG
15	wild	7.66	Not yet adult	2.5	6	0	JG
16	wild	7.76	Not yet adult	3.2	8	0	JG
17	wild	7.76	Not yet adult	2.6	9	0	JG
18	wild	10.03	Adult	2.6	6	0	JA
19	wild	10.10	Adult	2.9	2	0	JG
20	wild	10.81	Adult	2.3	6	0	JG
21	wild	11.29	Adult	2.9	7	0	JG
22	wild	13.23	Adult	3.0	13	0	JG
23	wild	13.86	Adult	3.0	9	0	JA
24	wild	14.19	Adult	2.0	6	0	JG
25	wild	14.29	Adult	2.4	15	0	JG
26	wild	14.47	Adult	3.1	9	0	JG
27	wild	15.72	Adult	1.9	16	0	JG
28	wild	7.25	Adult	3.1	3	0	JG
29	wild	7.40	Adult	3.2	14	0	JG
30	wild	7.40	Adult	2.9	1	0	JG
31	wild	7.43	Adult	2.8	2	0	JG
32	wild	7.57	Adult	2.9	11	0	JG
33	wild	8.15	Adult	2.9	11	0	JG
34	wild	8.21	Adult	2.9	3	0	JG
35	wild	8.29	Adult	3.2	3	0	JA
36	wild	8.34	Adult	3.2	1	0	JG
37	wild	8.37	Adult	3.4	3	0	JG
38	wild	8.79	Adult	2.3	4	0	JG
39	wild	8.89	Adult	2.9	6	0	JG
40	wild	9.19	Adult	2.8	1	0	JG
41	wild	9.33	Adult	2.9	2	0	JG
42	wild	9.55	Adult	2.9	4	0	JG
43	wild	9.56	Adult	2.6	6	0	JG
44	wild	9.60	Adult	3.2	10	0	JG
45	wild	9.69	Adult	2.7	5	0	JA
46	wild	9.79	Adult	3.2	12	0	JG
47	wild	10.02	Adult	3.0	1	1	JG
48	wild	11.00	Adult	2.4	6	1	JA
49	wild	11.00	Adult	2.6	5	1	JA
50	wild	13.25	Adult	2.9	3	1	JG
51	wild	14.99	Adult	2.4	9	1	JG
52	wild	15.00	Adult	2.3	10	1	JA
53	wild	15.73	Adult	2.0	10	1	JG
54	wild	18.75	Adult	3.1	11	1	JG
55	wild	20.45	Adult	2.2	14	1	JG
56	wild	8.00	Adult	3.0	1	1	JA
57	wild	8.36	Adult	3.3	1	1	JG
58	wild	8.56	Adult	3.1	4	1	JG
59	wild	8.66	Adult	2.5	3	1	JG
60	wild	8.70	Adult	2.4	2	1	JG
61	wild	8.81	Adult	3.6	1	1	JA
62	wild	9.15	Adult	2.9	2	1	JA
63	wild	9.23	Adult	2.1	2	1	JG
64	wild	9.49	Adult	3.4	1	1	JA
65	wild	9.68	Adult	3.1	3	1	JA
66	wild	9.71	Adult	2.9	3	1	JG
67	wild	9.84	Adult	3.4	6	1	JG
68	wild	9.85	Adult	3.3	3	1	JG
69	wild	10.51	Adult	2.7	2	2	JG
70	wild	10.66	Adult	3.5	3	2	JA
71	wild	10.66	Adult	2.8	4	2	JG
72	wild	11.23	Adult	3.2	4	2	JG
73	wild	11.99	Adult	3.0	7	2	JG
74	wild	13.52	Adult	2.5	1	2	JG
75	wild	16.94	Adult	1.6	8	2	JG
76	wild	17.99	Adult	1.1	15	2	JG
77	wild	19.51	Adult	0.6	6	2	JG
78	wild	8.63	Adult	3.6	1	2	JA
79	wild	9.88	Adult	2.7	1	2	JG
80	wild	11.07	Adult	3.0	5	3	JA

*Birthdate status indicates whether the birthdate is known or estimated, and what the error interval around the estimate is. 0 indicates an age known to within a few days; 1 indicates an estimate to within ± 1 year, 2 indicates within ± 2 years, and so on.

** ‘Researcher’ indicates who measured the canines: JA: Jeanne Altmann, and JG: Jordi Galbany.

### Immobilization of study subjects, acquisition of tooth casts, and canine measurements

We briefly immobilized the study subjects by propelling a dart from a blowpipe to deliver a dose of an anaesthetic, Telazol. This technique has been used successfully in this and several other primate populations (e.g., [34,37–41]). Upon immobilization, we observed and measured canine length. Canines varied in length and in sharpness; some were blunt, some were short but sharp, and some were obviously broken. In some cases, it is not possible to know if a canine tip was broken at some point, because after many years of wear, the break is difficult to see and may even become sharpen again through grinding. Consequently, in all cases, we measured the observable length of the canine, whether or not it appeared to be blunted through wear or was obviously broken.

The first subset of baboons was darted between 1989 and 1994. Canines for all subjects in this subset (*n* = 23) were measured directly by one observer (JA). Specifically, the upper left canines were measured using a metallic tape, with 1 mm of accuracy, from the tip to the gum at the distal side. The second subset of baboons was darted between 2006 and 2010 (*n* = 57), and tooth molds were made for all subjects for studies of tooth morphology and dental wear (see [35,42–43]). Tooth molds were obtained for the left mandibular and maxillary rows by washing the enamel tooth surfaces using a manual water pump, brushing lightly with a soft toothbrush to remove food remains, drying with a foot-powered air pump, and then using *Speedex* putty (Coltène/Whaledent Inc.—Cuyahoga Falls, Ohio, U.S.A.) to obtain an impression. The putty and the catalyzer were mixed rapidly and applied by hand, and pressed against all the teeth to insure high quality replicas. When the replication material was totally cured, the mold was removed, labeled, and stored in a plastic bag. From these negative molds, replicas were made at the University of Barcelona, using *Feropur* polyurethane (Feroca Composites S.A.—Madrid, Spain), which is stable, has excellent fluidity and has good hardness properties [35,44–45]. Upper left canines for each mold were then measured by one observer (JG) using the same methodology as in 1989–94, from the tip to the distal gum, using a metallic tape, with accuracy to the nearest 1mm. Using a tape measure with a resolution of 1 mm introduces potential error that could be minimized with calipers. However, we opted to use the tape measure so that we could analyze both the samples from 1989–1994 (the ‘JA’ sampling period; no casts exist for those teeth) and the samples from the most recent decade (the ‘JG’ sampling period). We concluded that doing so would reduce our accuracy somewhat but would not introduce systematic bias in our measurements. We also constructed a simple model (ANCOVA, α = 0.05) considering the sampling period (JA vs. JG), age, and the interaction of both as predictors of canine length in adult wild-feeding males. This model showed that the sampling period did not contribute significantly to variance in canine length (*p* = 0.193), which suggests that both observers measured the canines in a similar way. Data on canine length for all individuals are provided in Table 1.

### Statistical analyses

Our statistical analyses followed our three major hypotheses. First, we sought to compare canine development in food enhanced (*n* = 5) and wild-feeding (*n* = 12) young males in the Amboseli population in order to test Hypothesis 1, which predicts that a nutritionally enhanced diet would contribute to earlier eruption and hence longer-for-age canines relative to wild-feeding males. Specifically, we carried out two related analyses for this hypothesis. (i) We performed simple linear regression analyses, separately for food enhanced and wild feeding males, to assess the relationship between canine length and age in maturing males. (ii) We constructed a linear model including feeding condition (food enhanced vs. wild-feeding), age and the interaction of feeding condition and age as predictors of canine length in order to determine whether feeding condition was associated with significant differences between groups. For this analysis, we included all males less than 8 years of age. We considered males to have attained adulthood when they began to win agonistic interactions with adult males, which occurs at a median of 7.4 years in wild-feeding males in Amboseli [46–47].

Second, we quantified tooth wear in canines as a function of age, in order to test Hypothesis 2, which predicts that canines wear down as males age. In this analysis, we considered only fully adult males (those that had attained adult dominance rank). Further, this analysis included only wild-feeding males (*n* = 63), as we lacked sufficient data to carry out the analysis for adult food-enhanced males. Ages for subjects in this dataset ranged from 7.25 years to 20.5 years. We carried out both linear and quadratic regressions of canine length on age, in order to allow for the possibility that canines continued to elongate into early adulthood before beginning to wear down with age.

Finally, we determined the relationship between canine length and adult male dominance rank, which is a predictor of reproductive success in Amboseli and other populations [16,18,48–50], to test Hypothesis 3, which predicts that males with long-for-age canines achieve higher dominance rank. Again we used only fully adult wild-feeding males in this analysis. To do so, we first obtained the dominance rank for each male at the time of his immobilization. We obtained the residuals of the relationship between age and canine length from the quadratic regression described to test hypothesis (2) (*n* = 63), and used these residuals as a predictor variable, with dominance rank as a dependent variable. That is, we tested the hypothesis that males with relatively long canines for their age tended to be higher ranking. We note that the analysis of residuals can produce biased estimates of effect sizes [51–52]. However, in this case, our analysis is conservative, in that it may underestimate the effect of interest because true canine length effects could be masked by first taking into account the focal individual’s age. In other words, our analyses are likely to be biased towards type II error (false negative) rather than type I error.

All statistical tests were made with SPSS 15.0, MS-Excel for Windows, and SMATR (Standardized Major Axis Tests and Routines) software. P value threshold for significance was 0.05 in all cases.

## Results

### Hypothesis 1: Canine length during maturation

**Fig 1:**
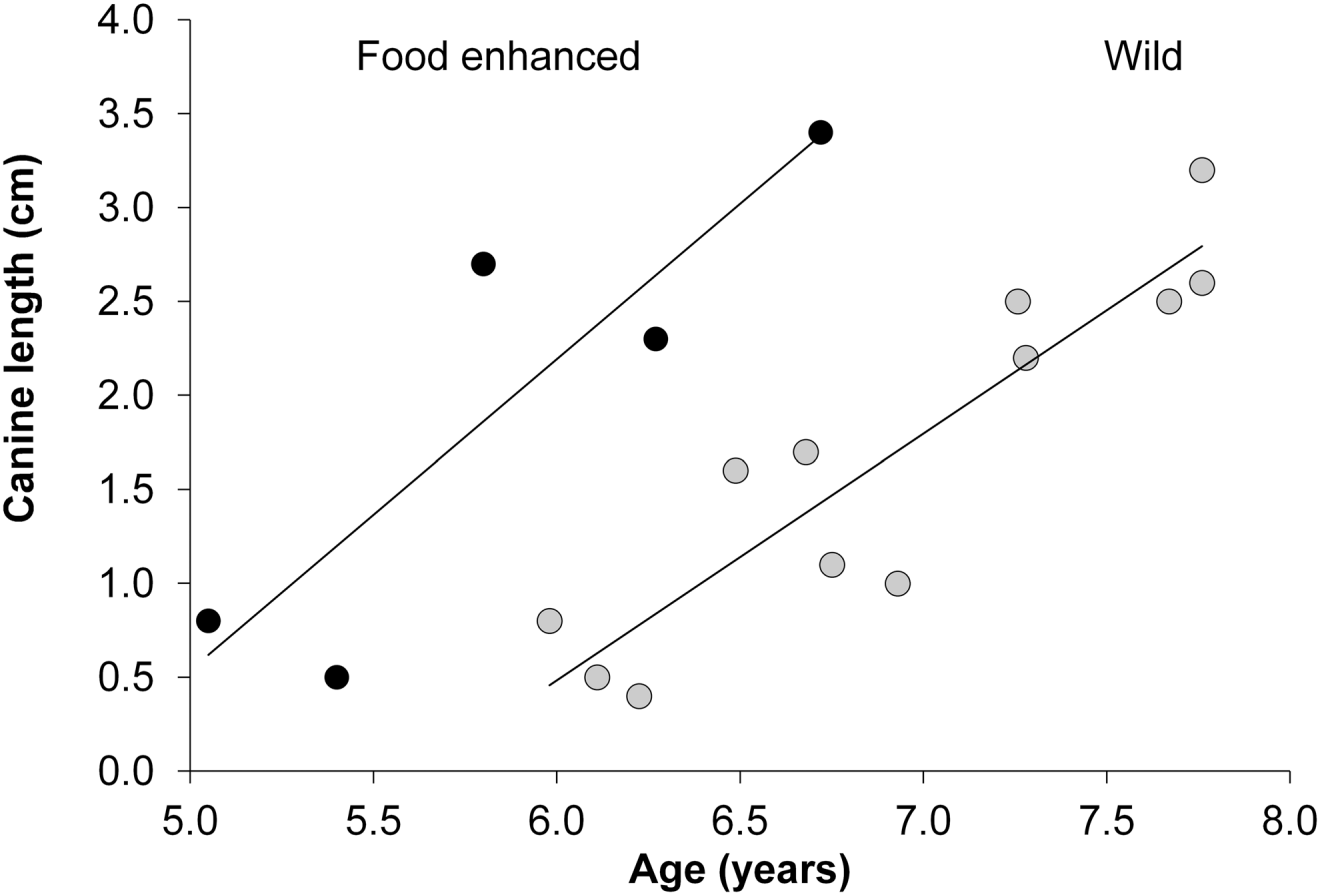
Canine length during maturation: linear regressions of canine length on age for wild-feeding and food-enhanced males who had not reached adulthood. In the equations, y is the response variable and R^2^ is the proportion of variance in the response variable that is associated with the predictor variable.

Canine length for both food-enhanced (*n* = 5) males and wild-feeding (*n* = 12) males between 5 and 8 years of age was significantly linearly correlated with age. The slopes of the relationship between canine length and age were similar across the two feeding conditions (Food enhanced canine length = 1.65*age - 7.71, F_1,3_ = 10.904, R^2^ = 0.784, *p*<0.05; wild feeding canine length = 1.31*age - 7.39, F_1,10_ = 48.120, R^2^ = 0.828, *p*<0.001) (Fig 1). However, the intercepts were significantly different between wild-feeding and food-enhanced males: a simple model considering feeding condition (food enhanced vs. wild-feeding), age and the interaction of feeding condition and age on canine length shows that feeding condition was associated with significant differences between groups (*p*<0.0001). The biological significance of this shifted intercept but similar slope is that food-enhanced baboons had much longer canines for a given age than wild-feeding baboons, not because the canines grew faster, or because they ultimately grew longer, but simply because they grew earlier. For example, wild-feeding males at age 6 years had a predicted canine length of 0.47 cm, while food-enhanced males of the same age had a predicted canine length of 2.19 cm.

### Hypothesis 2: Canine length during aging

The longest canines in our sample of wild-feeding adult males were 3.6 cm (in two males estimated to be 8.6 and 8.8 years of age), and the shortest was 0.6 cm (in a male estimated to be 19.5 years of age, whose canine appeared to be broken as well as heavily worn). To determine whether including animals with estimated ages affected our results for the relationship between canine length and age, we first performed independent regressions for the known-age males (n = 29) and the age-estimated males (n = 34) separately. First, we performed simple linear regressions. Both regressions were statistically significant (Canine length for known-age males = -0.073*age + 3.563, F_1,27_ = 8.876, R^2^ = 0.247, *p* = 0.006; vs. Canine length for estimated-age males = -0.124*age + 4.160, F_1,32_ = 25.036, R^2^ = 0.439, *p*<0.001), and the slopes of the two regression lines were not significantly different (Common slope = -0.170, SMATR T = 1.282, *p* = 0.259). Next, we performed quadratic regressions, to account for the possibility that canines continue to elongate into early adulthood. Again both regressions were statistically significant (Canine length for known-age males = -0.007*age^2^ + 0.085*age + 2.733, F_2,26_ = 4.519, R^2^ = 0.258, *p =* 0.021; vs. Canine length for estimated-age males = -0.005*age^2^ + 0.016*age + 3.291, F_2,31_ = 12.512, R^2^ = 0.447, *p*<0.001).

**Fig 2:**
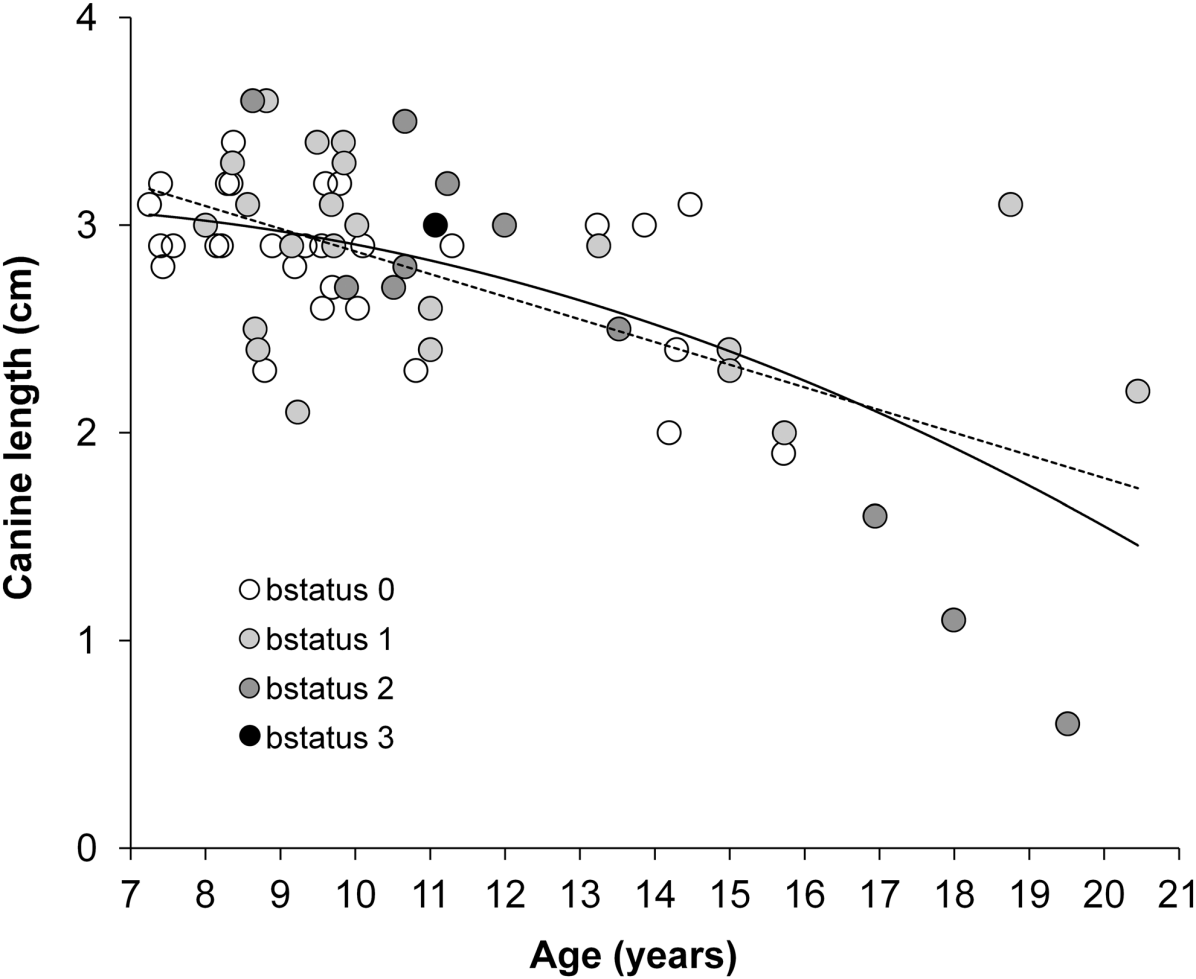
Canine length during aging: linear and quadratic regressions of canine length on age for wild-feeding adult males. In the equations, y is the response variable and R^2^ is the proportion of variance in the response variable that is associated with the predictor variable. ‘bstatus0’ indicates that the animals have an exactly known age; ‘bstatus1’ indicates that the age was estimated to within ± 1 year; ‘bstatus2’ indicates that the age was estimated to within ± 2 years, and so on.

Because we saw no difference in the age-related patterns of canine length in the known-aged and estimated-age males, we pooled the two sets to gain power in describing the general relationship between canine length and age. Cross-sectional analyses of canine length for all wild-feeding adult males (*n* = 63) revealed a significant relationship with age in both a quadratic regression (Canine length = -0.007*age^2^ + 0.080*age + 2.828, F_2,60_ = 20.931, R^2^ = 0.411, *p*<0.001, AIC = 17.45), and in a linear regression (Canine length = -0.107*age + 3.935, F_1,61_ = 38.751, R^2^ = 0.388, *p*<0.001, AIC = 15.58) (Fig 2). Wild-feeding males achieved their greatest canine lengths at around 8 to 9 years of age, and canine length declined at an accelerating rate thereafter (Fig 2). The delta AIC values for these two models was less than 2, indicating that they are both equally parsimonious. However, because the quadratic regression explained slightly more variance, we chose this one to pursue Hypothesis 3.

### Hypothesis 3: Canine length and social dominance rank

**Fig 3:**
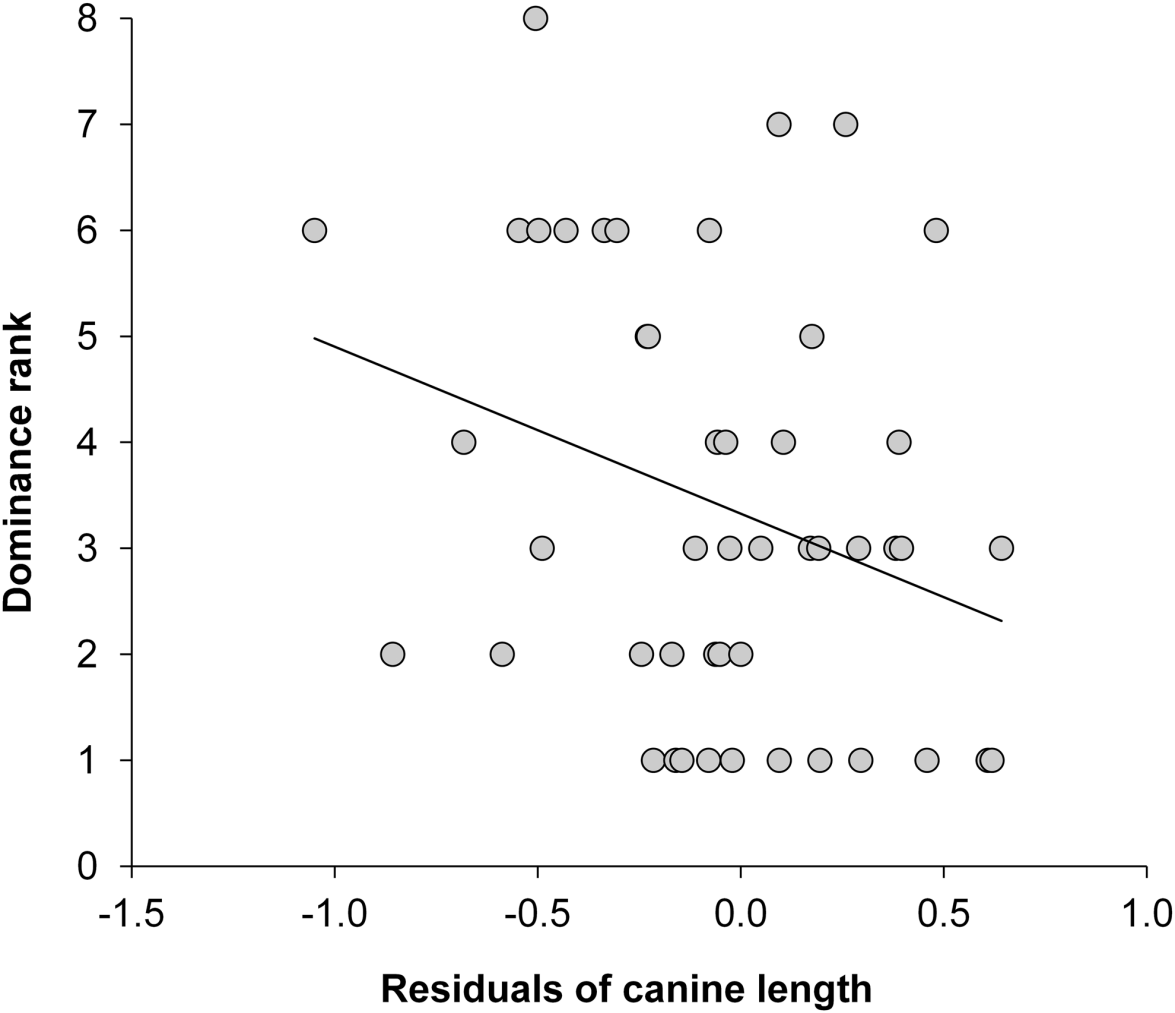
Canine length and social dominance rank: linear regression of canine length-for-age on social dominance rank, for males in the top half of the hierarchy, ranks 1–8. Length-for-age values were calculated as the residuals obtained in the quadratic regression of canine length on age. In the equation, y is the response variable and R^2^ is the proportion of variance in the response variable that is associated with the predictor variable.

We next examined the residual variance from the quadratic model linking canine length to age. Positive residuals indicated that a given baboon had long canines for its age, while negative residuals indicated an individual with short canines for its age; hereafter we describe these residuals as length-for-age. When all social dominance ranks were included in the analysis (from rank 1 to rank 16, *n* = 63), we found no significant linear relationship between dominance rank and length-for-age: Dominance rank = (0.23*length-for-age) + 5.54, F_1,61_ = 0.032, R^2^ = 0.001, *p* = 0.86. However, we found that canine length may influence dominance rank for higher ranking males. When we restricted our analyses to the top half of the ordinal rank distribution (ranks 1 through 8, *n* = 47, because lower ranking males rarely sire offspring) [16], we found a marginally significant linear relationship between dominance rank and length-for-age that explained ~9% of the variance in dominance rank: Dominance rank = (-1.59*length-for-age) + 3.33, F_1,45_ = 4.510, R^2^ = 0.091, *p*<0.05 (Fig 3). That is, among the top 8 ordinal ranks, males that occupied higher dominance ranks tended to possess longer canines for their ages than did males that occupied lower dominance ranks.

## Discussion

### Canine growth and diet

We sought to measure sources of variance in canine length in a population of wild baboons, and to evaluate the potential significance of canine length for male reproductive success. Our results support the hypothesis that the timing of canine growth in Amboseli baboons during maturation depends on nutrition. We showed that the enhanced diet consumed by males that fed partly on tourist refuse resulted in canines that were long-for-age compared to the canines of wild-feeding baboons, but that the canines appear to be elongating at the same rate in both sets, once erupted (Fig 1). Although we do not have data on the timing of canine eruption under the two different diet regimes in our study, our results are consistent with the idea that eruption occurred earlier in the food-enhanced males, and then proceeded at a similar rate to the rate experienced later by wild-feeding males (i.e., the slopes of the two regression lines were similar, but the intercepts were different). These results are in accord with a previous study done in captive and wild baboons, in which provisioned animals exhibited earlier tooth eruption than wild feeding animals [19]. The pattern of earlier tooth growth we report here is also consistent with the large differences in growth and maturation that have been associated with major differences in food availability in this and other primate populations and species (e.g., [21–22,24,53–57]). Moreover, in the Amboseli baboons, the differences in feeding ecology between food-enhanced and wild-feeding baboons have been associated with differences in infant survival, with infants in the food-enhanced group experiencing 27% higher survival than wild-feeding animals [33]. However, comparative analyses of periodontal health and of insulin and lipid concentrations in food-enhanced and wild-feeding baboons in Amboseli have indicated that animals that fed on tourist refuse also experienced poorer periodontal health and poorer metabolic profiles than wild-feeding animals [58–59]. In the aggregate, the fact that food-enhanced animals experienced accelerated growth and enhanced infant survival relative to wild-feeding animals, but reduced periodontal and metabolic health, suggests that eating abundant, calorically dense foods can involve a tradeoff between enhanced early survival and growth on the one hand, and compromised later life periodontal and metabolic health on the other.

### Canine length, age, and social dominance rank

Our cross-sectional analysis of canine length in adult male baboons suggests a dynamic history of change throughout the life course, as we have also found for the molar teeth [35,43]. We found a significant quadratic relationship between canine length and age for adult males, which explained ~41% of the variance in canine length. However, in spite of the strength of this relationship, considerable variance in canine length remained unexplained by age, making canine length alone a poor indicator of age. The unexplained variance in canine length no doubt resulted from the fact that canines suffer attrition from many causes over the life course. Enamel is lost during feeding, especially during the consumption of abrasives [35], and also when males open and close the mouth in canine displays or “yawns,” which sharpen the lower premolars and the upper and lower canines [25–26]. Male-male fights can blunt or break the canines, shortening them; predatory behaviors may also act as a potential source of canine damage [27,60–61]. Close to 28% of analyzed adult males in the present study showed at least one broken or blunt upper canine.

In spite of the substantial canine attrition we documented, adult male baboons typically possess large canines for a number of years during adulthood (from ~8 to 14 years of age; Fig 2). This period corresponds closely to the ages at which males sire the most offspring in this population (Fig 2 in [16]). Further, this period also corresponds to the ages at which males hold the highest dominance ranks [16,62]. The fact that canine length and dominance rank change in a similar manner with age makes it difficult to differentiate the effects of canine length from those of age on dominance rank, but it certainly suggests that canine length may affect fighting ability.

Our analysis of whether canine length predicted social dominance rank provided additional information relevant to this question. We examined the residuals of canine length after controlling for age, and found that canine tooth size in adult baboons may account for some of the residual variance in dominance rank that is not accounted for by age. Specifically, we found that higher-ranking males (those with ordinal ranks 1 to 8) had longer canines for their age. This relationship was not found when males with ordinal ranks lower than 8 were included in the analysis.

In other words, canine length may contribute to dominance rank, but its effects are only apparent for relatively high ranking males (those above the median rank in our data set). Three features of male baboon behavior and life history may explain these results. First, low-ranking males are generally older males, whose canines are likely to be short and thus relatively ineffective as weapons. Consequently, length differences in canines among older males may not translate into differences in how effective their canines are as weapons. Second, only higher ranking males are likely to successfully mate and reproduce, making weaponry more salient for these males than for older, lower-ranking males. Indeed, in a previous analysis of paternity success in Amboseli males, we found that males with ordinal ranks lower than 8 rarely sired offspring [16]. Consequently, among older, lower-ranking males, competition for dominance rank may be somewhat more relaxed and hence less dependent upon dangerous weapons. Third, it may be that canine sharpness is as important as canine length, and that a male’s skill or confidence in fighting also play a major role in determining dominance rank. If so, the effects of canine length on dominance rank would be modified by canine sharpness—a trait that we did not quantify—and by male fighting skill. That is, a short canine may be effective if it is sharp. These additional variables could explain, for instance, why the males that occupied rank 1 in our sample (the ‘alpha’ males) still exhibited substantial variation in canine length, with canines that ranged from 2.5 to 3.6 cm long (the range of canine lengths in all adult males in our sample was 0.6 to 3.6 cm; mean canine length in adults was 2.75 cm; see Table 1). Demographic variation among the groups in the male competitive environment may also explain why males of a given dominance rank still showed a range of canine lengths.

Nonetheless, our results are generally in accord with those found in mandrills [12], and support the hypothesis that canine length may play a role in determining social rank, in addition to other biological variables as body size or age [21]. Social rank can have striking effects on mortality and disease risk in primates because of its effects on immune function, physical condition, stress, reproductive effort, and testosterone levels [63]. In Amboseli baboons, low rank in adult males is associated with advanced age and with poor physical condition, and low-ranking males experience slower wound healing than high-ranking males [11]. The maintenance of high rank is energetically costly and depends on physical strength and condition. Males who are not in peak physical condition are unlikely to maintain high rank. Because canine length is both important for fighting ability, and inevitably decreases with age as a result of wear and tear, our results support the hypothesis that physical strength and condition drive dominance rank in male baboons.

More generally, our results provide strong general support for the hypothesis that sexual dimorphism in canine size is closely linked to male-male competition for access to reproductive opportunities. The evolutionary origins of sexual dimorphism in primate canine size have been much debated (reviewed in [7,9]). Our results join a growing body of evidence supporting a strong role for sexual selection in producing the marked sexual dimorphism in the canines of cercopithecine primates.
